# Back to the Future: The American Society of Tropical Medicine and Hygiene, Its Journal and the Continuing Commitment to Global Health

**DOI:** 10.4269/ajtmh.2011.10-0666

**Published:** 2011-01-05

**Authors:** James W. Kazura, Jonathan Ripp, Wilbur K. Milhous

**Affiliations:** Case Western Reserve University, Cleveland, Ohio; Mount Sinai School of Medicine, New York, New York; University of South Florida, Tampa, Florida

These are exciting and challenging times in the world of global health. The burden of major infectious diseases that disproportionately affect the health of women and children in low- and middle-income countries can, in some circumstances, be reduced substantially by implementing scientifically validated and evidence-based public health interventions, e.g., vaccination for diarrheal disease caused by rotavirus infection, insecticide-treated bed nets for malaria. Global efforts have been implemented that in the foreseeable future have as their stated goal local geographic elimination and ultimately, worldwide eradication of pathogens that historically have caused great suffering and socioeconomic loss in low- and middle-income countries. The paper by Linehan and colleagues^1^ in this issue of the *AJTMH* demonstrating the feasibility of an integrated approach to the elimination or control of five “Neglected Tropical Diseases,” lymphatic filariasis, onchocerciasis, schistosomiasis, soil-transmitted helminthiases, and blinding trachoma, is an outstanding example of how years of basic and clinical research can be translated into locally led national programs that make a real difference in the lives of people in developing countries. At the same time that these ambitious programs are unfolding, the underlying causes and demographics of ill health in the world are changing. Obesity, diabetes, and cardiovascular disease, once uncommon (or unrecognized) among poor people in developing countries, are increasingly common, especially in the megacities of Africa, Latin America, and Asia. Building on the prominence of “Global Health” at the November 2010 Annual Meeting of the American Society of Tropical Medicine and Hygiene, the Society's *Journal* will address global health education, research, and advocacy throughout the 2011 issues. Each month a global health article will be highlighted in the *AJTMH*. We welcome and will solicit perspectives and research papers on topics that pertain to global health advocacy, the metrics of competence in specific areas of global health education, health economics, collaborations that bridge infectious and chronic disease, and model public–private partnerships in developing countries.^2^ This represents a continued evolution of the Society and the *Journal* to “open the tent to all comers” and fulfill the byline of the Society: “Advancing global health since 1903.” Details and examples of articles that are sought are presented in the following paragraphs.

In May 2010, the Global Health interest group was officially chartered as the ASTMH Committee on Global Health (ACGH) with this mission: to provide a venue for presentation, dialogue, advocacy, and collaboration surrounding global health issues that are transnational, multidisciplinary, and disproportionately affecting underserved populations worldwide. The ACGH will aim to meet a number of goals including a high level global health research dissemination, education, career development, and pursuit of partnerships with like-minded organizations. The 59th Annual meeting was the first to have a Global Health pre-course. Given the interdisciplinary nature of the field, a case study approach was chosen as a backdrop to highlight the multiple approaches needed to address global health issues. The response to the January 2010 Haiti Earthquake was chosen as the timely and important global health “case.” The course featured a review of the abundant challenges that existed before the earthquake and how they were multiplied in the wake of a natural disaster. Representatives from governmental and non-governmental groups and the media generated considerable dialogue. Common themes included the outpouring of volunteerism which, despite the use of a “cluster” organizational approach, led to a considerable lack of coordinated efforts. Some controversy existed regarding the role of the media, although all were in agreement that this natural disaster created enormous devastation and tragedy in a country where dire conditions existed before the earthquake. Two additional symposia addressed the enormous health needs and lessons learned in the health response to the disaster in Haiti. Another symposium focused on careers in global health and included perspectives and insights from dynamic and inspirational global health practitioners. The group included primarily clinicians representing a range of careers including academic research, government, military, and academic education. Though the audience was quite engaged, some commented on the need to broaden the exposure to role models in global health research science, public health, and policy—a testament to the broad appeal of the Society's membership.

ASTMH proactively supports the growth of multidisciplinary groups to address the greater complexities of life sciences research and education, and advance economic development and global solutions. ASTMH recognizes that comprehensive education in global health is a necessary foundation on which to build these multidisciplinary collaborations. Faculty and students in global health programs have a vested interest in defining and engaging global health as an emerging health discipline. In support of our students and their future employers, ASTMH is working closely with the Association of Schools of Public Health to build a consensus on the core competencies for students in global health. Although resulting competencies will be applied to global health programs in schools of public health and other graduate institutions, the ultimate goal is to translate what is learned in the classroom into the field. With 108 years commitment to advancing global health, ASTMH is the flagship global health organization in the United States. Our membership is gaining a stronghold on who's doing what for which disease indications in virtually every corner of the planet. Our annual meetings and the *AJTMH* continue to serve as unprecedented venues to illustrate and promote the architecture and landscape for global health initiatives from academia, governments, industry, domestic, bilateral, and multilateral non-governmental organizations (NGOs).
